# Computed tomography colonography imaging of pneumatosis intestinalis after hyperbaric oxygen therapy: a case report

**DOI:** 10.1186/1752-1947-5-375

**Published:** 2011-08-15

**Authors:** Jean-Louis Frossard, Philippe Braude, Jean-Yves Berney

**Affiliations:** 1 Service of Gastroenterology and Hepatology, Geneva University Hospital, Rue Gabrielle Perret-Gentil 4, 1211 Genève 14, Switzerland; 2 Radiology Institute at Clinique de la Colline, Avenue de Beau Séjour 6, 1206 Genève, Switzerland; 3 Emergency Department, Geneva University Hospital, Rue Gabrielle Perret-Gentil 4, 1211 Genève 14, Switzerland

## Abstract

**Introduction:**

Pneumatosis intestinalis is a condition characterized by the presence of submucosal or subserosal gas cysts in the wall of digestive tract. Pneumatosis intestinalis often remains asymptomatic in most cases but may clinically present in a benign form or less frequently in fulminant forms. Treatment for such conditions includes antibiotic therapy, diet therapy, oxygen therapy and surgery.

**Case presentation:**

The present report describes the case of a 56-year-old Swiss-born man with symptomatic pneumatosis intestinalis resistant to all treatment except hyperbaric oxygen therapy, as showed by computed tomography colonography images performed before, during and after treatment.

**Conclusions:**

The current case describes the response to hyperbaric oxygen therapy using virtual colonoscopy technique one month and three months after treatment. Moreover, after six months of follow-up, there has been no recurrence of digestive symptoms.

## Introduction

Pneumatosis intestinalis (PI) is a condition in which submucosal or subserosal gas cysts are found in the wall of the small or large bowel [1]. PI may affect any segment of the gastrointestinal tract. The pathogenesis of PI is not understood but many different causes of pneumatosis cystoides intestinalis have been proposed, including mechanical and bacterial causes [2]. Whatever the pathogenesis, gas forming bacteria gain access to the submucosa through breaches in the mucosa and, once inside the bowel wall, gas may spread along the bowel and mesentery to remote sites. In most cases PI is an incidental finding, whereas in others PI is secondary to a wide variety of gastrointestinal and non-gastrointestinal diseases [3,4]. The true incidence of PI is not known but it is increasingly reported because of the more frequent use and improvement in imaging modalities. PI can be seen at any age but usually affects patients > 50 years old. PI usually remains asymptomatic in most cases but may clinically present in a benign form or less frequently in fulminant forms, the latter condition being associated with an acute bacterial process, sepsis, and necrosis of the bowel [1]. Symptoms include abdominal distension, abdominal pain, diarrhea, constipation and flatulence, all symptoms that may lead to an erroneous diagnosis of irritable bowel syndrome [5]. Complications of PI such as bowel obstruction, volvulus, pneumoperitoneum and hemorrhage occur in about 3% of patients [1]. Treatment for PI includes antibiotics, elemental diets, surgery and oxygen therapy. Here we report the case of a patient who responded very well to hyperbaric oxygenotherapy.

## Case presentation

**Figure 1:**
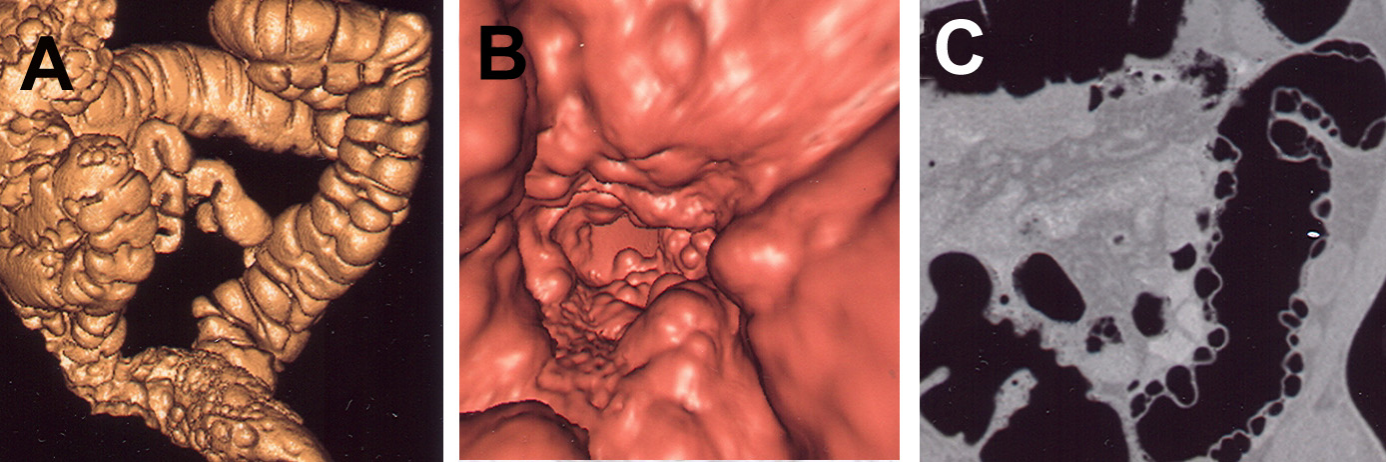
**(A) Oblique view of surface rendering computed tomography colonography (CTC) image demonstrating multiple gas cysts in the left colon and sigmoid**. **(B) **Fly-through view of CTC of the sigmoid. **(C) **Coronal view of CTC showing multiple submucosal poylpoid nodules on the wall of sigmoid.

A 56-year-old Swiss-born man who had recently developed abdominal pain and changes in his bowel habits underwent an optical colonoscopy. He was otherwise in good health and had no medical history other than a tendency to constipation. Colonoscopy revealed multiple submucosal polypoid lesions covered with normal mucosa in the colon. An abdominal computed tomography (CT) scan with a soft tissue window setting did not show any abnormal findings in the abdomen. CT colonography identified multiple submucosal gas cysts mostly located in the left colon (Figure 1A-C).

**Figure 2:**
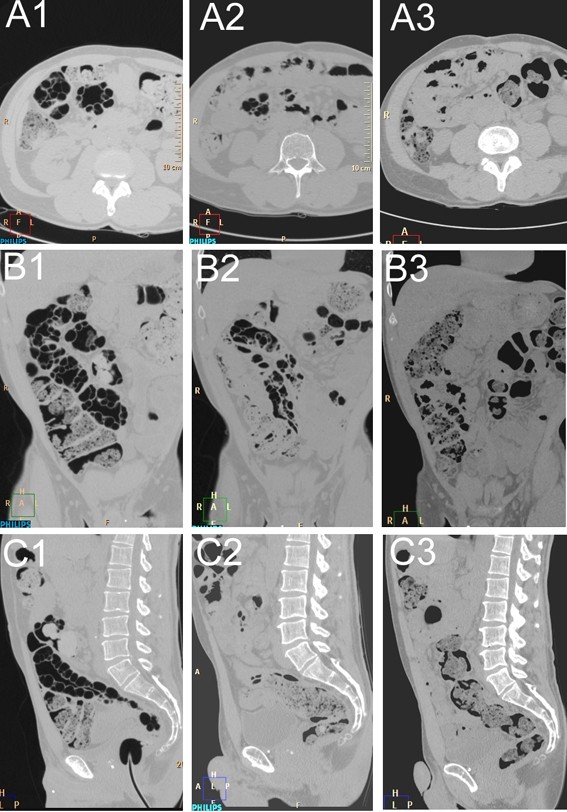
**(A1-3) Axial view**. (A1) Images taken before treatment. (A2) Images taken six weeks after treatment. (A3) Images taken 12 weeks after treatment. Note the almost complete disappearance of the cysts within the colonic wall. **(B1-3) **Coronal view taken at the same time schedule as (A1-3). **(C1-3) **Sagittal view taken at the same time schedule as (A1-3).

Initially our patient was given nutrition advice and was administered painkillers as well as a moderator of bowel transit, which were unsuccessful. Several attempts at antibiotic treatment were made to help decrease the size of the cysts, but without success. Before recourse to surgery because of untractable symptoms, our patient was given hyperbaric oxygen therapy (HBO_2_), which is one of the treatment options for patients with symptomatic PI. Three sessions were performed on non-consecutive three days, two using table Comex 30 (heliox 50/50) and one at 2.5 atmospheres absolute (ATA) for 90 minutes, with transient relief lasting a couple of weeks. We therefore decided on additional sessions for 14 days, a Comex 30 session followed daily by 90 minutes at 2.5ATA, which resulted in a decrease in cyst size and an almost complete return to normal bowel anatomy as depicted in Figure 2. Moreover, our patient was completely asymptomatic at six-month follow-up.

## Discussion

Pneumatosis intestinalis is an uncommon condition in which multiple gas-filled cysts are located in the wall of the colon, as shown in our patient's case. The multiple thin-walled, non-communicating, gas-filled cysts may be found in either the submucosal or subserosal layer of the gastrointestinal wall [2]. Three different possible sources of gas within the intestine are considered nowadays: intra-luminal gas, gas produced by bacteria and pulmonary gas. For the latter, the possibility of gas coming from the lungs as a source of PI is debated but is mainly based on the theory of air migration along the vessels within the mediastinum, retroperitoneum and mesentery after alveolar rupture in pulmonary diseases [6]. Thankfully most patients are asymptomatic, but they may present with various clinical conditions including vomiting, intestinal distension, abdominal pain, diarrhea or constipation [2].

Treatment options for PI include antibiotics, elemental diets, and oxygen therapy, whereas surgery is restricted to patients who remain symptomatic despite medical therapy or when they present with life-threatening conditions such as bowel obstruction or hemorrhage. To date there have been no controlled studies aimed at evaluating the effect of antibiotics in this condition, but the disappearance of PI after a course of antibiotics may support its efficacy, as it is reported in a previous case series [4]. Numerous reports have shown the success of metronidazole in treating patients who are symptomatic, with a typical course of 500 mg orally three times daily [7].

Inhalation oxygen therapy was first introduced in the early in order to increase the partial pressure of oxygen in the blood and to decrease the partial pressure of non-oxygen gases in the cysts [8]. This change in oxygen delivery in the cysts is supposed to create a diffusion gradient across the cystic wall that will finally result in the exit of gas from the cysts. Numerous reports have shown the success of inhalation oxygen therapy, but the optimal amount and duration of such treatment to induce cyst deflation is not known. HBO_2 _is also successful in treating PI and has the major advantage of avoiding the pulmonary toxicity of oxygen that can be associated with use of prolonged high flow oxygen [9]. The use of heliox seems to be more effective than O_2 _[10].

Whereas conservative treatment should always be considered as the first option, patients with increased inflammatory parameters in laboratory findings or signs of sepsis, peritonitis or bowel obstruction or perforation in combination with PI should undergo an explorative laparotomy [6]. It is worth mentioning that inappropriate surgery may worsen PI and deteriorate the general condition of the patient [6].

## Conclusions

Treatment options for PI include antibiotics, diet therapy, oxygen therapy and more invasive techniques such as surgery. The current report demonstrates the complete response to HBO_2 _therapy using a virtual colonoscopy technique one month and three months after treatment. Moreover, after six months of follow-up, there has been no recurrence of digestive symptoms in our patient. Therefore HBO_2 _should be considered in indicated cases and if the technique is available before considering more invasive therapeutic options such as surgery.

## Consent

Written informed consent was obtained from the patient for publication of this case report and any accompanying images. A copy of the written consent is available for review by the Editor-in-Chief of this journal.

## Competing interests

The authors declare that they have no competing interests.

## Authors' contributions

JLF, PB and JYB analyzed and interpreted the data from our patient regarding the CT colonography and evolution after treatment. JLF and JYB were major contributors in writing the manuscript. All authors read and approved the final manuscript.
